# Design of an Estimator Using the Artificial Neural Network Technique to Characterise the Braking of a Motor Vehicle

**DOI:** 10.3390/s22041644

**Published:** 2022-02-19

**Authors:** María Garrosa, Ester Olmeda, Vicente Díaz, Mᵃ Fernanda Mendoza-Petit

**Affiliations:** 1 Department of Mechanical Engineering, Universidad Carlos III de Madrid, Avda. de la Universidad 30, 28911 Madrid, Spain; eolmeda@ing.uc3m.es (E.O.); vdiaz@ing.uc3m.es (V.D.); mmendoza@ing.uc3m.es (M.F.M.-P.); 2 Institute for Automotive Vehicle Safety (ISVA), Universidad Carlos III de Madrid, Avda. de la Universidad 30, 28911 Madrid, Spain

**Keywords:** pressure sensor, artificial neural network, types of braking, brake pressure estimation

## Abstract

Automatic systems are increasingly being applied in the automotive industry to improve driving safety and passenger comfort, reduce traffic and increase energy efficiency. The objective of this work is focused on improving the automatic brake assistance systems of motor vehicles trying to imitate human behaviour but correcting possible human errors such as distractions, lack of visibility or time reaction. The proposed system can optimise the intensity of the braking according to the available distance to carry out the manoeuvre and the vehicle speed to be as less aggressive as possible, thus giving priority to the comfort of the driver. A series of tests are carried out in this work with a vehicle instrumented with sensors that provide real-time information about the braking system. The data obtained experimentally during the dynamic tests are used to design an estimator using the Artificial Neural Network (ANN) technique. This information makes it possible to characterise all braking situations based on the pressure of the brake circuit, the type of manoeuvre and the test speed. Thanks to this ANN, it is possible to estimate the requirements of the braking system in real driving situations and carry out the manoeuvres automatically. Experiments and simulations verified the proposed method for the estimation of braking pressure in real deceleration scenarios.

## 1. Introduction

Over the last decades, there have been major advances in automotive safety systems, both active and passive. Since the appearance of the Anti-lock Braking System (ABS) in the early 1970s, which was the first driver assistance system, new means have emerged to help reduce the likelihood of an accident or to reduce its consequences in case of an accident. The most important active safety equipment in vehicles is the braking system, which is a fundamental aspect of vehicle dynamics. The introduction of ABS was a major breakthrough for the automotive industry and a great improvement in safety as it assists vehicle braking in low-grip conditions.

The current breakthrough in the automotive industry is due, in terms of safety, to the continuous improvement of intelligent driver assistance systems. This boom is the result of manufacturers’ awareness and their aim to build ever safer vehicles to reduce the number of road fatalities. The latest are Advanced Driver Assistance Systems (ADAS), which aid the driver while on the road and thus improve the driving experience [1,2,3]. These systems interact with the driver to help him practice safer driving, taking control of the vehicle if necessary [4,5,6]. ADAS systems manage simple tasks, such as reducing the vehicle’s fuel consumption [7,8,9,10,11], and more critical manoeuvres too, like avoiding risky situations and collisions on the road by directly acting on others vehicle systems such as steering or brakes [12,13,14]. Among the most frequent road accidents is the head-on collision. This type of accident is caused by late and untimely driver intervention, as well as insufficient braking torque in emergency situations. Since many of these accidents are due to driver inattention and human error, progress in these assistance techniques enhances passenger safety and comfort. These assistance mechanisms include stability control, automatic braking, adaptive cruise control, hill descent control and traction systems. All these examples require onboard sensors, procedures for estimating variables, control algorithms and components that act according to the output of the sensors, so that the tasks for which they are intended can be carried out.

In the braking system of a standard motor vehicle, the braking torque is generated by the hydraulic pressure applied in the brake cylinder. Therefore, the precise measurement of the braking pressure by means of a pressure sensor and the estimation of this pressure is very important to characterise the braking performance of a vehicle. There is a multitude of research based on braking pressure observation methods [15,16,17,18,19]. In Reference [20], a recursive least square algorithm for brake cylinder pressure estimation was proposed to provide useful information to ABS. In Reference [21], an algorithm for estimation of the pressure in the wheel cylinder based on the extended Kalman filter was developed, considering the hydraulic model and tyre dynamics. In Reference [22], a hydraulic model and inverse model were developed based on the characteristics obtained from the simulation of the active pressure system. The active pressure system included the control valves, eccentric rotary plunger pump, brake pipe/hose and throttles. The models can be used to the inline estimation with Electronic Stability Program (ESP) and obtain the wheel brake pressure. In Reference [23], a brake pressure estimation algorithm for ABS was proposed considering the hydraulic fluid characteristics. In Reference [24], the braking pressure estimation algorithm was carried out by calculating the volume of fluid flowing through each hydraulic valve. In Reference [25], a braking system architecture based on the use of proportional servovalves for continuous brake pressure control was proposed. The optimum pressure to be applied to each wheel was obtained from a fuzzy logic control block. In Reference [26], a probabilistic estimation method of brake pressure was developed for electrified vehicles based on multilayer artificial neural networks with Levenberg–Marquardt backpropagation. In Reference [27] an integrated time series model based on multivariate deep recurrent neural networks with long short-term memory units was developed for the dynamic estimation of the brake pressure of electrified vehicles. In Reference [28], an adaptive sliding mode hydraulic pressure controller based on a hydraulic pressure estimator to track desired hydraulic pressure for “sensorless” electrohydraulic brake system was proposed. In Reference [29], a double closed-loop cascade control architecture with interconnected pressure estimation for a pressure-sensor-unequipped integrated electrohydraulic brake system was presented. In Reference [30] an algorithm based on vehicle information considering the evolution of the brake lining’s coefficient of friction was proposed for the estimation of the master cylinder pressure of the electrohydraulic brake system.

In vehicle behaviour prediction, Long Short-Term Memory (LSTM) are the most widely used deep models [31]. To predict the intention of vehicles, LSTM is used in References [32,33,34] as a sequence classifier. However, in Reference [35], it is shown that, in some driving scenarios, feed-forward neural networks can have competitive results with faster processing time compared to LSTMs. The large processing time of LSTMs is due to the fact that they are sequential and cannot be parallelised. In addition, LSTMs are vulnerable to parameter tuning and need task-specific engineering, such as gradient clipping. These drawbacks do not appear in feed-forward networks. Feed-forward neural networks are computational models capable of learning, storing and retrieving information based on a training dataset. ANNs are useful for solving many engineering problems that are difficult to tackle using conventional methods. They are very flexible and can be used generally to learn a map from inputs to outputs. ANN are suitable for classification prediction problems where inputs are assigned a class or label and for regression prediction problems where a value is predicted given a set of inputs.

The problem presented in this study, the estimation of vehicle braking, does not have long dependencies, and the input data does not form a sequence. For this reason, a feed-forward network has been chosen instead of LSTM.

Motivated by the previous review, this article provides an innovative design of an estimator of the pressure in the hydraulic circuit of the braking system and the type of braking manoeuvre: maintained, progressive or emergency.

The contributions of this work with respect to the existing approaches are:

1. The proposal of a novel methodology for the analysis of the data acquired by the sensors during the experiments. New indicators were defined in order to characterise the braking manoeuvre of a vehicle, providing information on type of braking, intensity or evolution over time.
2. The development of an ANN-based estimation algorithm to estimate the pressure in the brake circuit and the type of braking. The system was implemented with the experimental data obtained from the sensors during the experiments. Therefore, the system will brake by imitating human behaviour.
3. The proposed braking system automatically decides how to apply the brake when faced with the risk of a collision. It achieves this by using the information obtained by the sensors about the obstacle. Depending on the position of the obstacle and the speed of the vehicle, the actions on the braking system to reduce the speed will be to perform (1) maintained, (2) progressive and (3) emergency braking. In other words, the automatic braking offers safe and comfortable brake control, without braking too early or too late.

The manuscript is organised as follows: Section 2 explains the vehicle instrumentation and the methodology employed to carry out the experiments. Section 3 shows the measurement of the sensors and describes the methodology applied for the analysis of the data obtained in the experimental tests. Section 4 presents the different ANN architectures that have been designed, and the results of the simulations and their validation are included in Section 5 and Section 6, respectively; the results are discussed in Section 7, and Section 8 concludes the paper.

## 2. Materials and Methods

**Figure 1:**
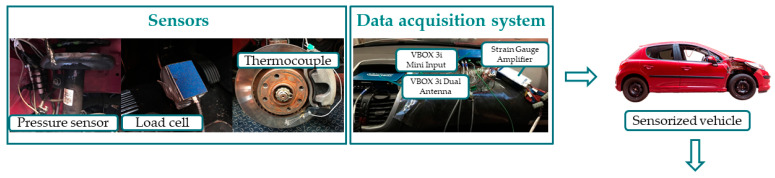

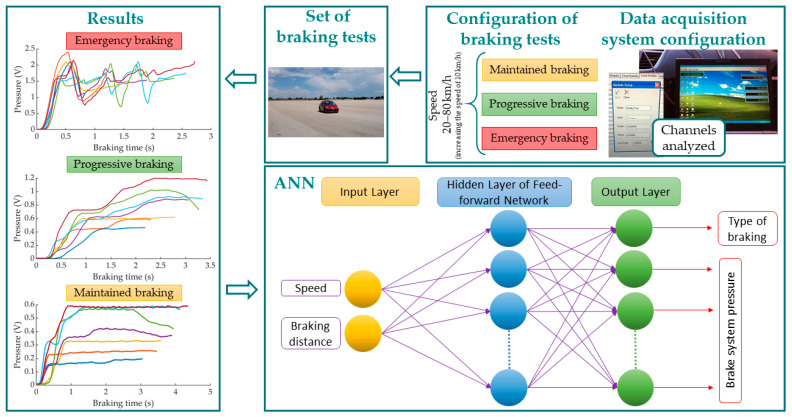
Methodology followed for the development of the study presented in this article.

Figure 1 shows schematically the flow diagram for the development of the research presented in this paper. A vehicle was instrumented with different sensors and a series of braking tests were carried out. The real results collected by the sensors during the experiments allowed the design of an ANN-based estimation system that simulates these results in order to characterise braking of any nature and enables it to be used in real traffic conditions.

The instrumentation used in this study is explained in Section 2.1, and the methodology that has been carried out for the proper development of the experimental phase is detailed in Section 2.2.

### 2.1. Instrumented Vehicle

**Figure 2:**
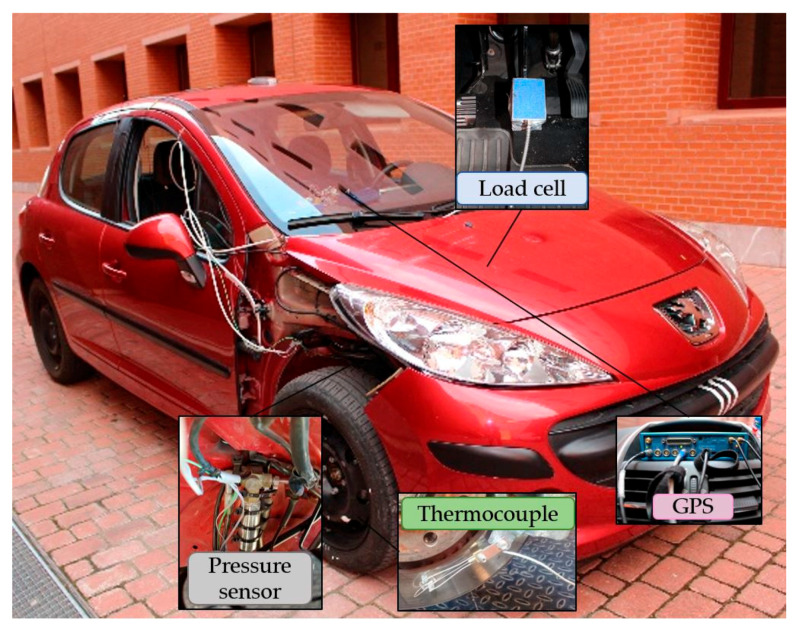
Instrumented vehicle.

For the experiments, a Peugeot 207 1.6 HDI 16v was instrumented with two pressure sensors incorporated in the independent hydraulic circuits of the front wheels, a load cell installed in the brake pedal, a thermocouple in the front right brake disc and a Global Positioning System (GPS) receiver, as shown in Figure 2. The sensors are described in detail in the next subsections.

#### 2.1.1. Pressure Sensors

**Figure 3:**
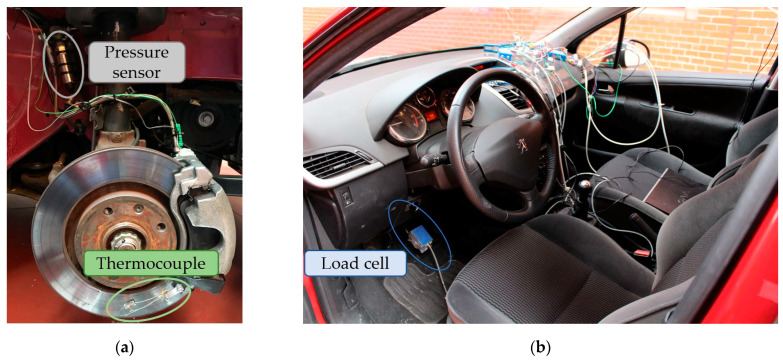
Onboard sensors: (**a**) pressure sensor in the brake circuit and thermocouple on the brake disc and (**b**) load cell on the brake pedal.

The vehicle’s braking system was equipped with strain gauge pressure transducers in both of the front hydraulic circuits in order to know the instantaneous pressure during the tests. The control of the pressure in the brake circuit is an indicator to determine when to limit the pressure in the circuit as a means of altering the braking capacity of the vehicle. These sensors convert the pressure into an electrical signal by deforming the four strain gauges in the diaphragm inside the sensor. The pressure applied to the sensor causes a deflection of the diaphragm, which flexes the gauges, causing a measurable voltage difference proportional to the pressure at the point of study. The pressure sensors were installed between the brake calliper inlet and the last section of the hydraulic brake circuit, as shown in Figure 3a. The sensors are of the DRUCK LIMITED brand, type PDCR 911 and with an operating range from 0 to 135 bar.

#### 2.1.2. Thermocouple

The brake disc was instrumented with a K-type thermocouple to measure the temperature reached during the tests (see Figure 3a). The brand of the thermocouple is TC DIRECT, with mineral insulation of 0.5 mm in diameter. The temperature range is from 0 to 850 °C, and the time constant is 0.03 s.

#### 2.1.3. Load Cell

A load cell was fixed on the braking pedal to determine exactly when the driver starts the braking manoeuvre. The beginning of measurement of this sensor was the trigger for the data collection of the different sensors instrumented in the vehicle. The device is the HKM PK 2.0 (see Figure 3b) with an operating range from 0 to 1500 N.

#### 2.1.4. Data Acquisition System

The data acquisition equipment used in the experimental phase was the VBOX 3i data logger with Dual Antenna. The data logger uses a GPS/GLONASS receiver. The most relevant characteristics that were considered when selecting this equipment are: the sampling frequency (up to 100 Hz), the ease of assembly and transport, the ability to accept both analogue and digital inputs and outputs, the visualization of results in real time on a computer thanks to a USB or Bluetooth connection and its low power consumption.

**Figure 4:**
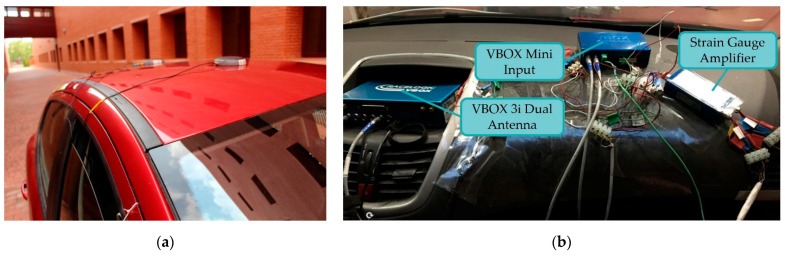
(**a**) Twin antennas located longitudinally on the roof of the vehicle and (**b**) vehicle dashboard data acquisition equipment.

Two antennas on the vehicle roof in line with the direction of motion (see Figure 4a) were connected to the VBOX 3i and provided time, speed and position values.

The Mini Input Module (RLVBMIM01) was used to measure the sensor signals. The temperature sensor is connected to one of the K-type thermocouple inputs and the pressure sensors and the load cell to the analogue inputs. To condition the signal from the two pressure transducers, it was necessary to install Racelogic’s Strain Gauge Amplifier (RLVBSGA01), which is designed for full Wheatstone bridge operation. Figure 4b shows the data acquisition equipment on the vehicle dashboard.

The VBOX 3i equipment has its own software (VBOX Tools) that allows to control all the modules involved in the measurements, manage the tests carried out in real time and store the obtained data.

### 2.2. Methodology of the Experimental Phase

In order to gather the necessary data to train the feed-forward neural networks, tests were carried out with the instrumented vehicle. These were performed thanks to the participation of fourteen drivers. The volunteers were men between the ages of 22 and 30. The scenarios consisted of reproducing a series of braking manoeuvres at different speeds on a flat track following a straight path. The tests distinguish between three types of manoeuvres with the aim of reproducing all the types of braking that can occur while driving a vehicle: maintained, progressive and emergency braking. These manoeuvres are explained in detail in Section 2.2.1. The test speeds were 20, 30, 40, 40, 50, 60, 60, 70 and 80 km/h. The driver drove the vehicle forward until the test speed was reached, and once the test speed was constant, the braking process was initiated until the vehicle stopped. Combining the type of braking and the test speed, each driver performed 21 experiments. Therefore, a database of almost 300 experiments was available for the study presented here. Table 1 shows the tests performed by each driver.

All tests were performed on a 300 × 250-m asphalt track on the facilities of the Instituto Nacional de Técnica Aeroespacial (INTA).

A sampling frequency of 100 Hz was set up for all data acquisition. This frequency can register with enough data resolution the signal from the different onboard sensors without losing information. All data collected during tests were stored to be analysed later.

#### 2.2.1. Types of Braking Performed in the Experimental Tests

As discussed previously, a driver can perform three types of braking to slow or stop the vehicle. Therefore, the following types of braking are defined in this study:

- Maintained braking

This type of braking is achieved by lightly pressing the brake pedal and holding it on this position until the vehicle stops. This behaviour can occur when the braking manoeuvre is predictable and the braking distance is long.

- Progressive braking

In order to achieve progressive braking, the driver has to progressively press the brake pedal until the vehicle stops. The braking force increase linearly over time. This type of manoeuvre is the most common in a real driving scenario.

- Emergency braking

Emergency braking occurs when the braking distance is reduced, and the manoeuvre is not expected. The driver must depress the brake pedal quickly to the end of its travel to stop the vehicle in the smallest possible distance. This type of braking corresponds to emergency cases.

**Figure 5:**
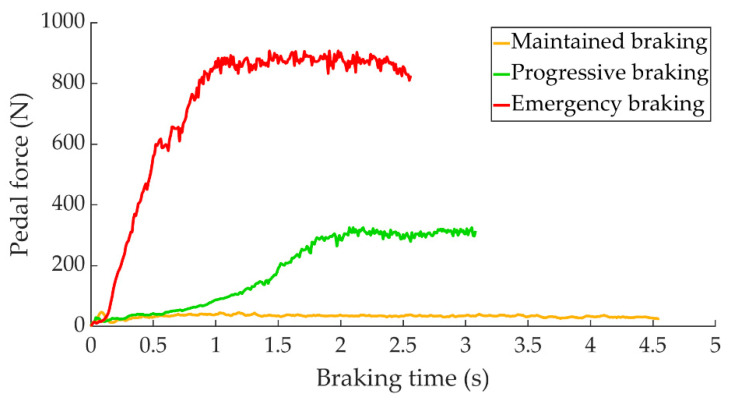
Force exerted on the brake pedal as a function of braking time for the three types of braking studied.

Figure 5 shows how the driver presses the brake pedal for each of the three types of braking.

#### 2.2.2. Variables Analysed in the Experimental Tests

The variables measured during the experimental tests are described below:

1. Braking time

When the driver presses the brake pedal, the installed load cell determines the start of braking as a “trigger”. The braking time ends when the GPS determines that the vehicle speed is equal to zero.

2. Braking distance

Thanks to the positioning of the GPS signal, it is possible to determine the distance travelled by the vehicle during the braking time.

3. Pressure in the brake circuit

Pressure sensors located near the brake calliper system measure the pressure in the hydraulic brake circuit.

#### 2.2.3. Test Conditions

The following boundary conditions were considered during the tests to ensure their repeatability:

- Tyre pressure should be within the manufacturer’s recommended range for the vehicle’s load.
- The temperature range allowed on the brake disc before each braking manoeuvre must be between 18 and 31 °C.
- There shall always be a second person in the co-driver’s seat in charge of controlling the acquisition system. No other persons are allowed in the vehicle.
- The clutch must be disengaged to avoid the influence of engine retention in braking capacity.

## 3. Data Collected by Sensors

The results obtained in the experimental tests are analysed in this section. The data are evaluated according to the type of braking proposed and the different test speeds. The results are structured in three sections. Section 3.1 provides the measurement data of the pressure sensors during the experiments. Section 3.2 contains a statistical study of all the recorded data. Section 3.3 explains the methodology for analysing the data collected by the pressure sensors.

### 3.1. Output Signal of the Pressure Sensors Installed for the Driving Braking Tests

This section shows some examples of the data obtained during the different braking tests by the pressure sensors. Thanks to this data, it is possible to analyse the influence of the type of braking and the test speed. These data are expressed in volts (V) and are not processed.

**Figure 6:**
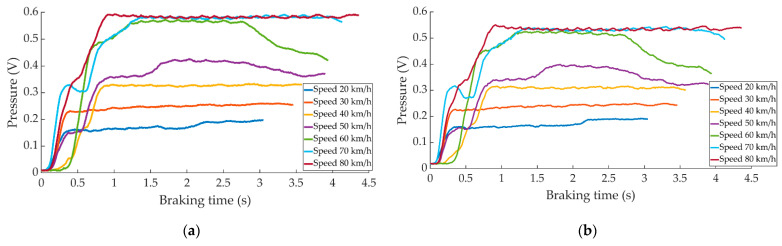
Curves of the data obtained during maintained braking for the different speeds by (**a**) the right pressure sensor and (**b**) left pressure sensor.

The Figure 6 shows a comparison of the pressure curves obtained during maintained braking for the different test speeds.

**Figure 7:**
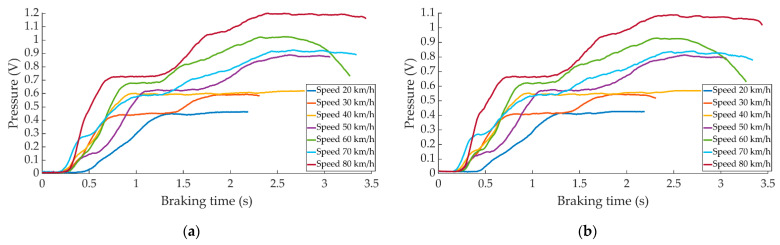
Curves of the data obtained during progressive braking for the different speeds by (**a**) the right pressure sensor and (**b**) left pressure sensor.

Figure 7 shows the effects produced by the speed variation on the pressure curves in progressive braking.

**Figure 8:**
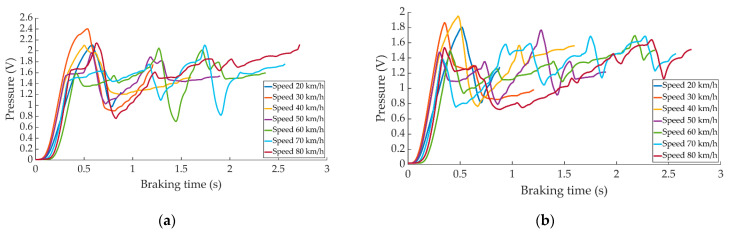
Curves of the data obtained during emergency braking for the different speeds by (**a**) the right pressure sensor and (**b**) left pressure sensor.

The pressure curves acquired in emergency braking as a function of the different test speeds are shown in Figure 8.

### 3.2. Statistical Study of the Driver Set

The following tables show information of the 14 drivers in every test speed (maximum, minimum, average and standard deviation of the variables measured during the experiments). The data related to maintained braking is shown in Table 2, the data related to progressive braking in Table 3 and, finally, the data related to emergency braking in Table 4.

Table 2, Table 3 and Table 4 show how the average of all signals increases as the test speed increases. This happens for the three proposed types of braking. For the same test speed, the highest values recorded by the pressure sensors are given for emergency braking, followed by progressive braking and the lowest values correspond to the maintained braking. Emergency braking is the fastest, followed by progressive braking and maintained braking is the slowest. This data is directly related to the braking distance required for each of the braking types.

### 3.3. Methodology for Analysing Data Collected by Pressure Sensors

An intelligent braking system must be able to obtain information from the different sensors on board the vehicle, process the data obtained from them and transform them into useful information for the active control of the vehicle in real time.

In order to study the almost 300 experimental tests that were carried out and to be able to use the data recorded during these tests in the ANN systems proposed in Section 4, new concepts have to be defined. These concepts are indicators used to characterise each of the braking manoeuvres performed. The procedure to define these new indicators is explained below.

**Figure 9:**
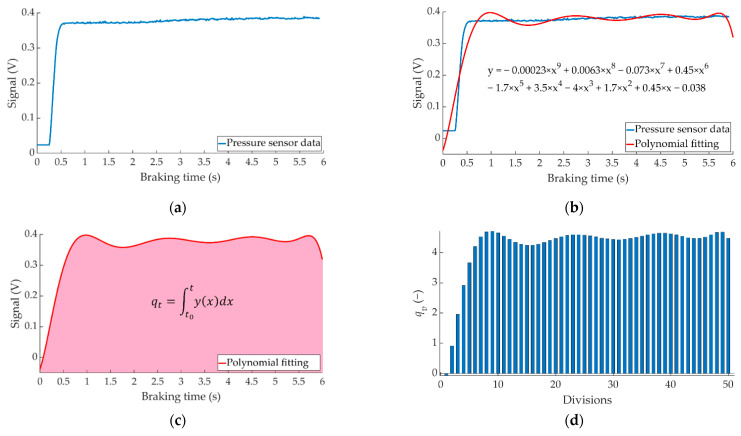
Methodology for the analysis of the data acquired by the pressure sensors to obtain the indicators. (**a**) raw sensor measurement, (**b**) fitted function, (**c**) *q_t_* and (**d**) *q_v_* for 50 divisions.

The first step is to fit to a polynomial the curves representing the time evolution of the real measurements obtained by the pressure sensors during each braking manoeuvre (see Figure 9a).

Knowing the fitted function (see Figure 9b), it is possible to calculate the area under the curve, “*q_t_*”. This indicator defines the integral of the fitted function from the beginning of the braking (*t*_0_) to the end of the braking (*t*) and provides significant information about the magnitude of the braking (see Figure 9c), but it is also important to know how the braking is distributed over time.

To know how the braking is distributed over time, the total braking time is divided into a fixed number of divisions. By calculating the area under the fitting curve for each division made, it is possible to have a representation of how the total measured magnitude is distributed. This information is stored in a vector called “*q_v_*” (see Figure 9d).

**Figure 10:**
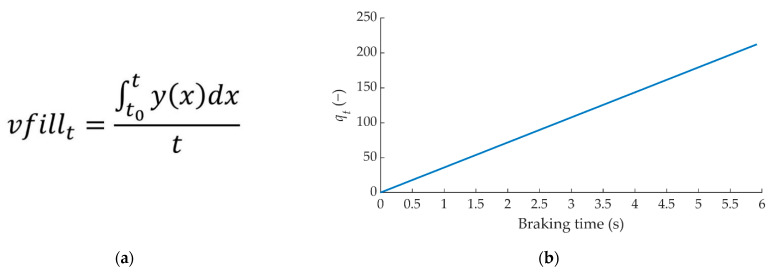

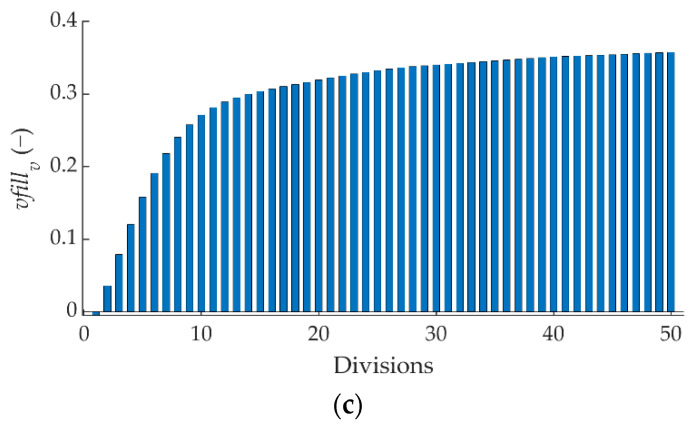
Methodology for the analysis of the data acquired by the pressure sensors to obtain (**a**) *vfill_t_*, (**b**) *q_t_*/*t* and (**c**) *vfill_v_* for 50 divisions.

If “*q_t_*” is divided by the time taken to execute the braking, the indicator called “*vfill_t_*” is obtained (see Figure 10a). This factor is an indicator of the total braking intensity (see Figure 10b).

As before, if “*vfill_t_*” is integrated by divisions as a function of time, the time representation of the braking evolution is obtained. This concept is a vector called “*vfill_v_*” and provides information on the braking intensity for each division (see Figure 10c). Each value of this vector represents the intensity of braking over time.

**Figure 11:**
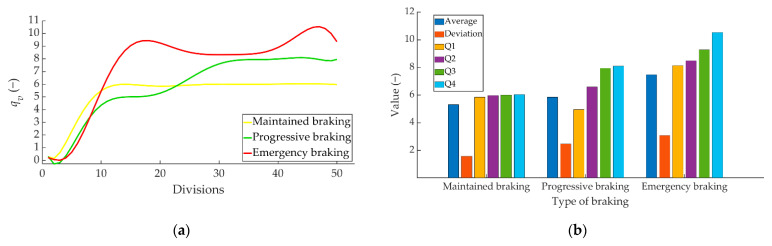
Example of the three types of braking at 80 km/h performed by one of the drivers (**a**) *q_v_* and (**b**) statistical data.

The following statistical data have been calculated from “*q_v_*” vector: average, standard deviation and quartiles. Figure 11a shows, by way of example, how the vector “*q_v_*” obtained for the same driver evolves at a speed of 80 km/h for the three types of braking. Figure 11b shows the statistical values obtained from the data represented in Figure 11a. The average and standard deviation values provide information on the magnitude of the braking. Q1, Q2, Q3 and Q4 provide information on the evolution of braking over time.

These statistical values were used as target data defining desired network output in training in addition to the indicators: *q_t_*, *q_v_*, *vfill_t_* and *vfill_v_*.

## 4. Feed-Forward Neural Networks

To transform the data collected by the different sensors into useful information for the vehicle’s active control systems, an ANN-based intelligent control model is proposed in this study.

ANNs are computational models capable of learning, storing and retrieving information based on a training dataset. Through training, a target output is achieved from a given input by modifying the weights, which are the values of the connections between the neurons that form the network. Depending on the problem to be solved, there are different network structures. In this case, the objective is to obtain the pressure in the brake circuit, as well as the type of braking (outputs) as a function of speed and braking distance (inputs). A two-layer feed-forward structure was chosen, as this scheme is quite efficient for function fitting.

The steps for ANN analysis are (1) pre-processing the database, (2) defining the input neurons, (3) defining the hidden layer of the feed-forward network and the estimation layer, (4) defining the loss function, (5) training the model, and (6) validating the model. Details of the above steps are given in Section 4.1.

### 4.1. ANN Model

This section justifies the decisions made during the implementation of the ANN models presented in this study. MATLAB’s Neural Network Toolbox has been used to model the neural networks.

Pre-processing the input and targets dataset is a common first step in the deep learning workflow to prepare raw data in a format that the network is able to accept. Therefore, before submitting the data to the network, they were normalised. In this study, the data have been normalised to speed up the model convergence process and increase the accuracy of the final model by using the “mapminmax” function in MATLAB. This function processes matrices by assigning the minimum and maximum values of the rows to [−1, 1]. The calculation equation is as follows:

(1) $$y=\frac{\left(y_{max}-y_{min}\right)\cdot \left(x-x_{min}\right)}{x_{max}-x_{min}}+y_{min}$$

where *y* is the processed data, *y_max_* = 1 and *y_min_* = −1, *x* corresponds to the original data to be normalised, and *x_max_* and *x_min_* are the maximum and minimum values of the original data to be processed, respectively.

To convert the network output into the original units once the network has been trained with the processed data, the “mapminmax_reverse” function is used.

The Multi-layer Perceptron Neural Network architecture (MLP) with backpropagation algorithm was chosen because of its ability to interpret and interpolate data, create relationships between input and output parameters and its ease of use and versatility. Choose the layers in which the neurons are distributed in the architecture of a MLP is a fundamental aspect. There are three types of layers: input, output and hidden. The characteristics of the input and output layers depend on the problem to be solved, since the number of neurons required in each of these layers is determined by the nature of the input and output patterns to be estimated. The hidden layer contains the neurons responsible for approximating nonlinear functions, thus linking the input and output layers. The number of hidden layers and neurons in each layer affect the capacity of the model for generalization and have to be empirically determined.

In this study, the input layer is composed of two neurons that correspond to the input variables of the designed system: longitudinal speed and braking distance. The reason that justifies this decision is based on the equipment of the vehicles currently on the market, which incorporate sensors that can obtain this information thus avoiding the installation of new sensors. Speed is a data that is easy to know at any time for any vehicle; in the same way, and thanks to the detection systems fitted in current cars, it is possible to obtain information on the distance between the front of the vehicle and other point (object on the road, another vehicle, etc.). This second value corresponds to the space available for the vehicle to stop.

The objective of the proposed ANN is to obtain the value of the pressure in the brake circuit, which is the output variable of the system; in addition to being able to characterise the type of braking manoeuvre that the vehicle has suffered (maintained, progressive or emergency). To optimise the performance of the ANN, output layers with different numbers of neurons are tested. The cases studied include 37, 57, 97, 137, 177 and 217 neurons in the output layer according to 5, 10, 20, 20, 30, 40 and 50 divisions in the pressure sensor data that define *q_v_* y *vfill_v_* parameters. The neurons in the output layer contain information about the type of braking and defined indicators for right and left pressure sensors in Section 3.3 (*q_t_*, *q_v_*, *vfill_t_*, *vfill_v_* and statistics).

ANN models are designed with one hidden layer that contains a different number of neurons: 10, 20, 30, 40 and 50.

The network has a sigmoid transfer function in the hidden layer and a linear transfer function in the output layer.

The output function taken for all neurons is the identity, so that the output signal of each neuron matches its own activation state.

**Figure 12:**
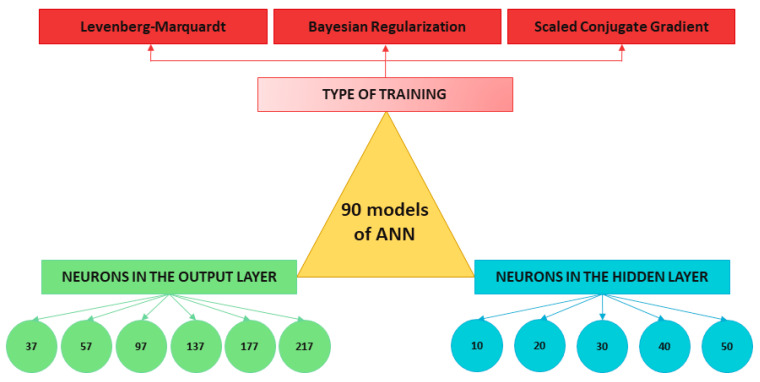
Design decisions for the development of ANN architectures.

The training of the ANN is also an important choice. In combination with the number of divisions and the number of neurons in the hidden layer, three different types of training are proposed: Levenberg–Marquardt (LM), Bayesian Regularization (BR) and Scaled Conjugate Gradient (SCG) (see Figure 12).

The data were randomly split into training, validation and test sets: 70% for training, 15% to validate that the network is generalizing and to stop training before overfitting and 15% to independently test network generalization. The training of the network is optimised using the default criterion used by the MATLAB Neural Network Toolbox, which consists of the minimisation of the Mean Square Error (MSE)—the average squared error between the network outputs and the target outputs (loss function). According to this, the smaller the MSE, the better the data is adapted. The main objective during the model construction process has been to maximise the regression value (R^2^) while minimising the MSE. R^2^ measure the correlation between the outputs and targets.

**Figure 13:**
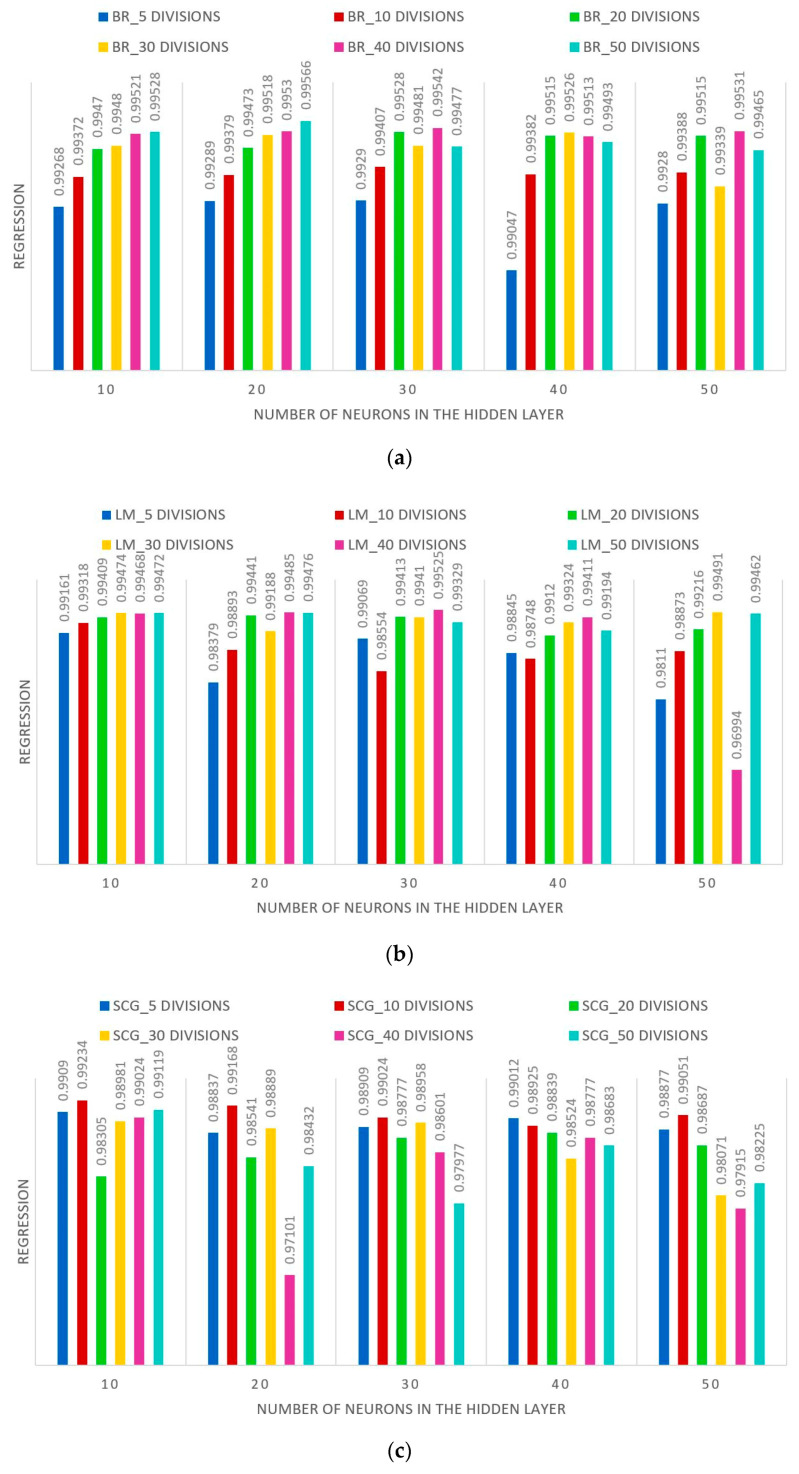
ANN sensitivity of the different models designed for the training type: (**a**) BR, (**b**) LM, and (**c**) SCG.

Combining the number of neurons in the output layer, number of neurons in the hidden layer and the type of training, 90 different network models have been trained to find out which one produces the best fitting results between inputs and targets. Figure 13 shows the influence of the different parameters mentioned on the sensitivity of the ANN according to the degree of regression of the input values with those returned by the system in its testing process (regression expressed over 1).

**Figure 14:**
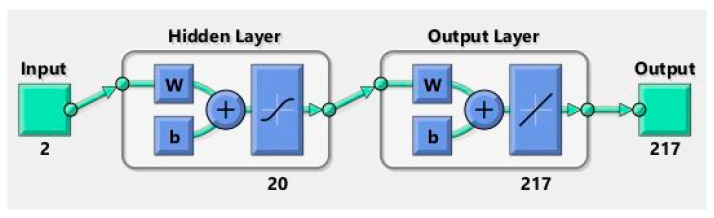
Architecture of the chosen ANN.

It has been observed that, in all models, when the MSE decreases, the R^2^ increases. Furthermore, the MSE decreases as the number of divisions performed in the output data vector increases. However, there is no relationship between the number of neurons in the hidden layer and the MSE. The best of the three algorithms tested was BR, followed by LM and, lastly, SCG. All models have a high degree of regression, however, and given that the computational cost is negligible (network training is not performed in real time, only the simulation and parameter estimation process take place in real time), an ANN with the following parameters has been chosen (see Figure 14).

- Number of neurons in the input layer: 2.
- Number of neurons in the hidden layer: 20.
- Number of neurons in the output layer: 217.
- Type of training: Bayesian Regularization.
- Divisions of the data vectors: 50.

The 217 neurons that make up the output layer correspond to:

- Position 1: Represents the type of braking that has been performed (maintained = 1, progressive = 2 or emergency = 3). It is contemplated that decimal values appear in position 1 of the output vector.
- Position 2: Represents the braking capacity value measured by the right pressure sensor (*q_t_*).
- Positions 3–52: Vector dividing by 50 the braking capacity value measured by the right pressure sensor according to the time the vehicle takes to stop (*q_v_*).
- Position 53: Represents how the right pressure sensor reaches full braking capacity, providing information on “how braking occurs over time” (*vfill_t_*).
- Position 54–103: Vector dividing by 50 the value of *vfill_t_* relative to the right pressure sensor (*vfill_v_*).
- Positions 104–109: Statistical values for braking characterisation relating to the right pressure sensor.
- Position 110: Represents the braking capacity value measured by the left pressure sensor (*q_t_*).
- Positions 111–160: Vector dividing by 50 the braking capacity value measured by the left pressure sensor according to the time the vehicle takes to stop (*q_v_*).
- Position 161: Represents how the left pressure sensor reaches full braking capacity, providing information on “how braking occurs over time” (*vfill_t_*).
- Positions 162–211: Vector dividing by 50 the value of *vfill_t_* relative to the left pressure sensor (*vfill_v_*).
- Position 212–217: Statistical values for braking characterisation relating to the left pressure sensor.

**Figure 15:**
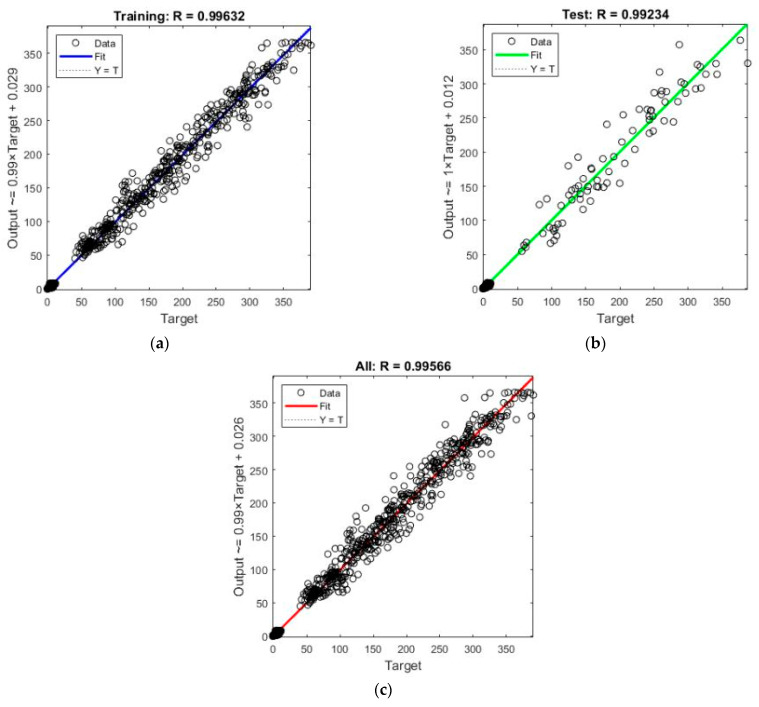
Regression obtained: (**a**) training phase, (**b**) test phase and (**c**) all system.

The training was stopped at iteration 874 when the learning rate reached the predefined value (5 × 10^10^). This indicates that the network convergence is correct. The value of R obtained for the training and test phase is shown in Figure 15. As a combination of these phases, the R value obtained for the total system is 0.99566 (expressed over 1).

## 5. Results of Braking Parameter Estimation

This section shows the results obtained after the different simulations of the chosen ANN. These values are compared with the parameters with which the system has been trained in order to check the correlation between the target values and the simulated values. Random simulations have been carried out covering the whole range of test speeds in combination with the different types of braking manoeuvres: maintained, progressive and emergency.

For a better understanding of the results, as an example, those relating to braking of the three types at a test speed of 70 km/h are shown.

**Figure 16:**
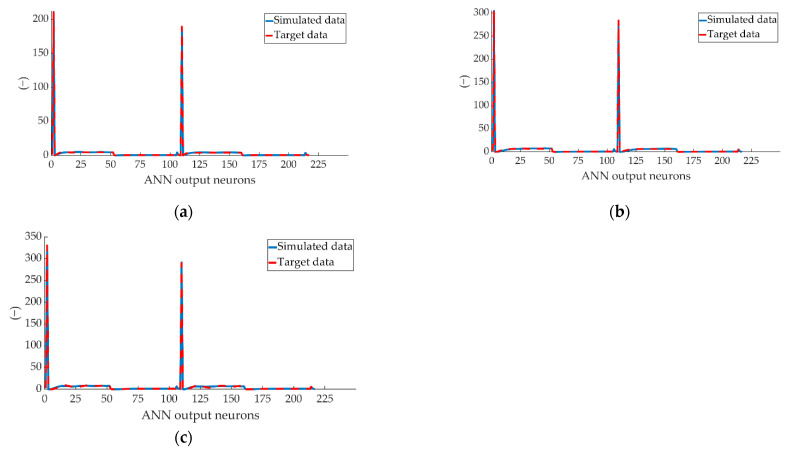
Comparison between the complete data simulated by ANN and the target data for braking at a speed of 70 km/h for type: (**a**) maintained, (**b**) progressive and (**c**) emergency.

Figure 16 shows the comparison between the simulation of the total values estimated by ANN and the total target data. The total values coincide with the parameters: *q_t_*, *vfill_t_*, *q_v_*, *vfill_v_* and statistical values of the two pressure sensors, as well as the type of braking.

As can be seen in Figure 16, the lines representing the simulations made from the estimated values and the target data practically overlap, a sign of an ANN with a high degree of fit and convergence. It is important to note that, due to the nature of the data used in the system, the output layer is made up of a vector of variables that take different numerical values. Due to this, when dealing with the results, the units of measurement of each independent variable have not been taken into account, giving importance to the interpretation of the data as a numerical value and interpreting each one by its position.

**Figure 17:**
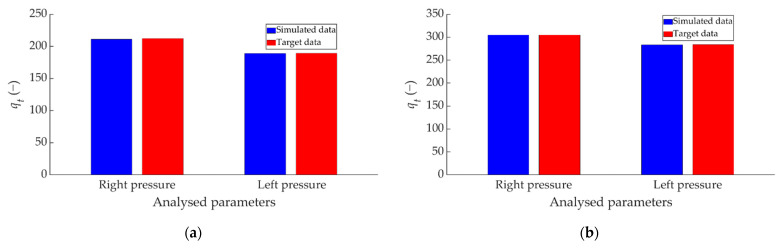

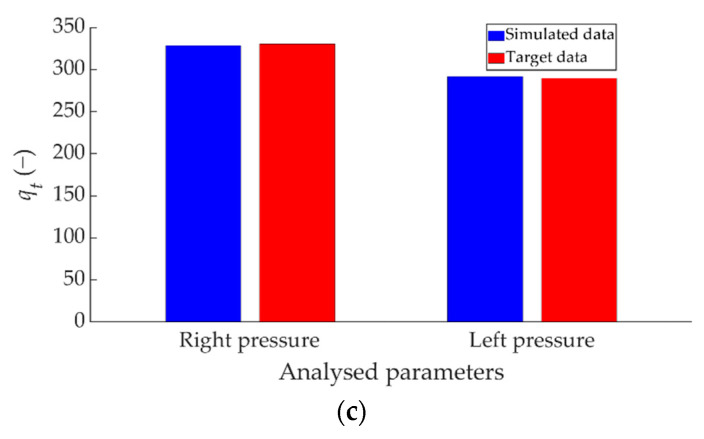
Comparison between ANN-simulated data and target *q_t_* data for the two pressure sensors for braking at a speed of 70 km/h for the type: (**a**) maintained, (**b**) progressive and (**c**) emergency.

To study the results independently, Figure 16 is split to analyse each dataset separately. Figure 17 compares the simulation of the ANN estimated values and the target data for the braking capacity (*q_t_*) for the two pressure sensors (right and left).

**Figure 18:**
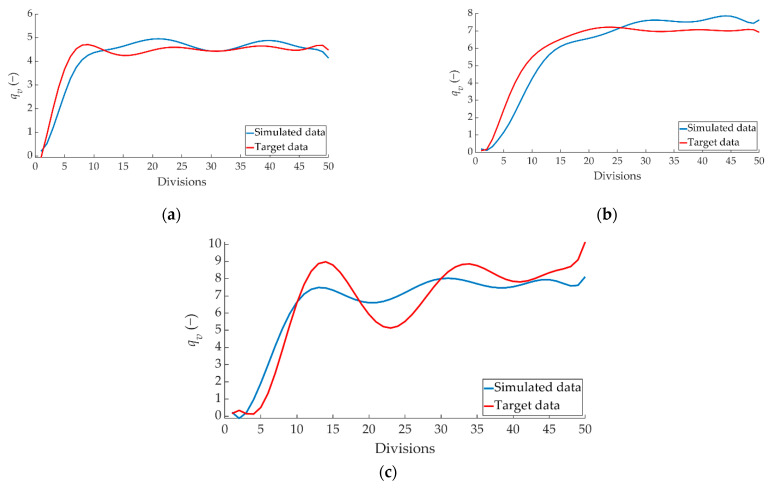
Comparison between ANN-simulated data and target *q_v_* data for the right pressure sensor for braking at a speed of 70 km/h for the type: (**a**) maintained, (**b**) progressive and (**c**) emergency.

Figure 18 shows the comparison between the simulated data and the target data on how the braking capacity evolves as a function of braking time (*q_v_*) for the right pressure sensor over the 50 divisions performed.

**Figure 19:**
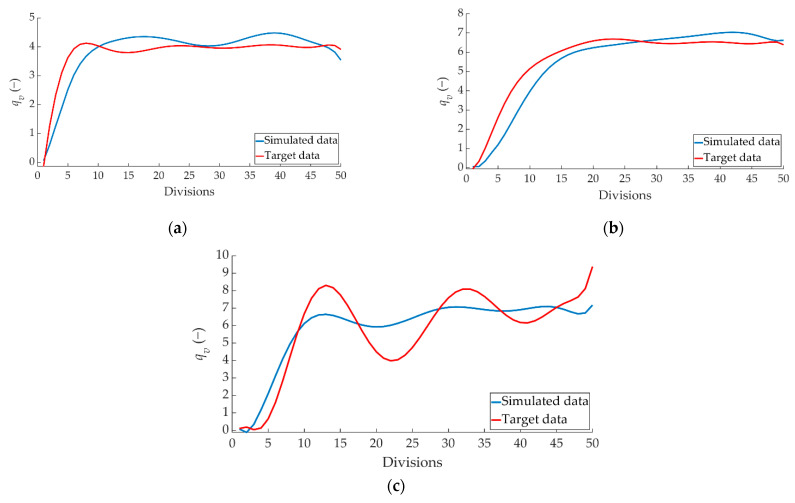
Comparison between the ANN-simulated data and target *q_v_* data for the left pressure sensor for braking at a speed of 70 km/h for the type: (**a**) maintained, (**b**) progressive and (**c**) emergency.

Figure 19 shows the comparison between the simulated data and the target data concerning the evolution of *q_t_* over the 50 divisions performed for the left pressure sensor.

**Figure 20:**
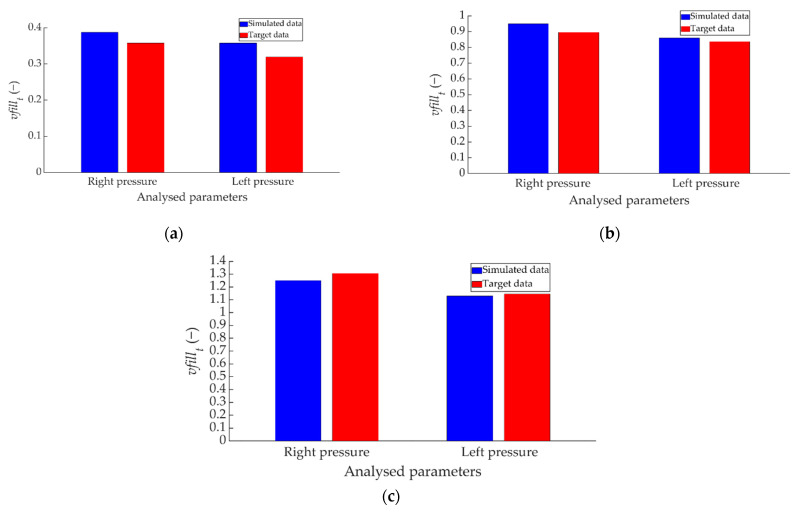
Comparison between the ANN-simulated data and *vfill_t_* target data for the two pressure sensors for braking at a speed of 70 km/h for the type: (**a**) maintained, (**b**) progressive and (**c**) emergency.

Figure 20 shows the comparison between the ANN-estimated values and the target data for *vfill_t_*.

**Figure 21:**
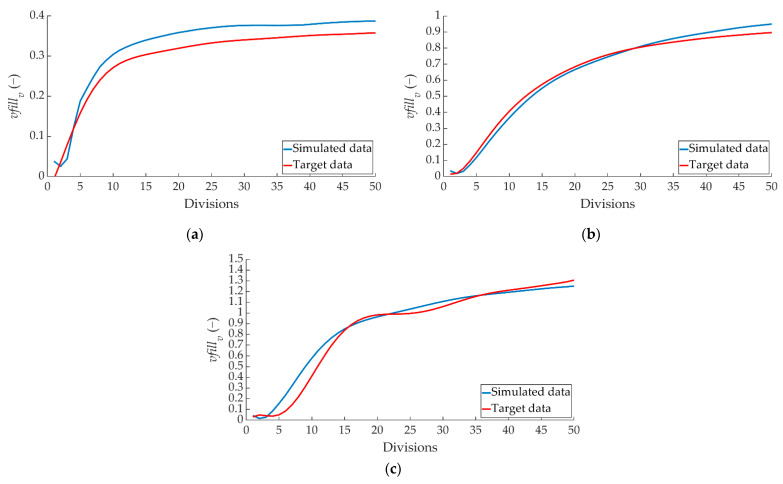
Comparison between ANN-simulated data and *vfill_v_* target data for the right pressure sensor for braking at a speed of 70 km/h for the type: (**a**) maintained, (**b**) progressive and (**c**) emergency.

Figure 21 shows the comparison between the ANN-simulated data and the target data for how the right pressure sensor achieves braking capacity as a function of braking time (*vfill_v_*).

**Figure 22:**
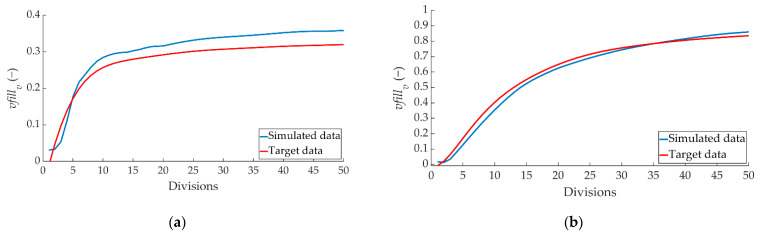

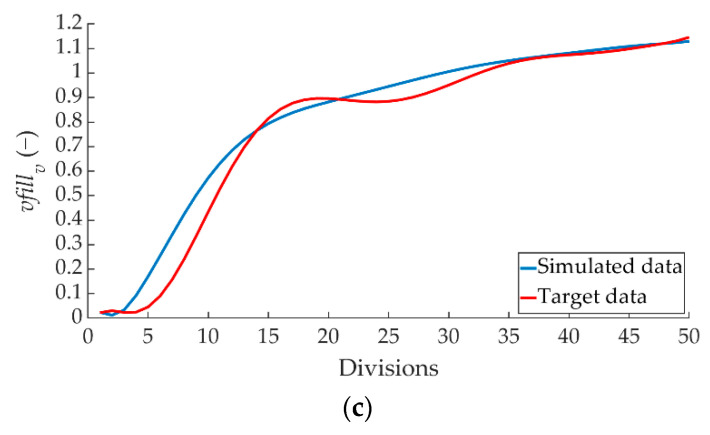
Comparison between ANN simulated data and *vfill_v_* target data for the left pressure sensor for braking at a speed of 70 km/h for the type: (**a**) maintained, (**b**) progressive and (**c**) emergency.

Figure 22 shows the comparison between the ANN simulated data and the target data for the *vfill_v_* parameter corresponding to the left pressure sensor.

**Figure 23:**
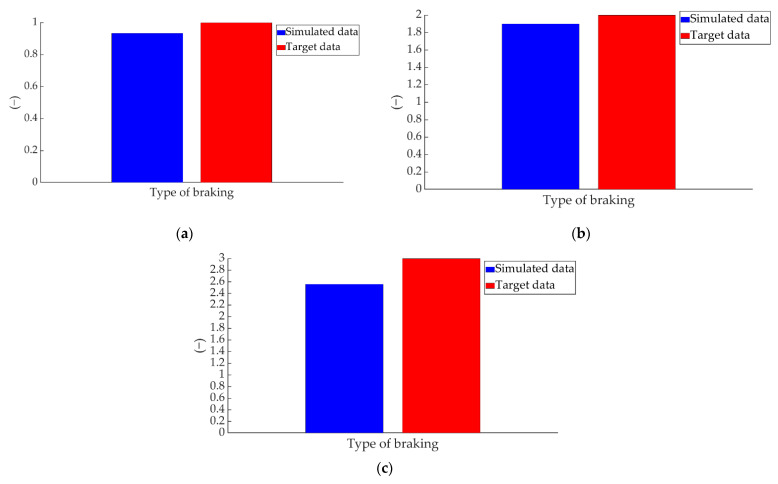
Comparison between the ANN-simulated data and target braking type data for braking at a speed of 70 km/h for the type: (**a**) maintained, (**b**) progressive and (**c**) emergency.

Another of the simulated data refers to the type of braking performed. The comparison between the simulated values and the target data of the braking type parameter for the three types of braking is shown in Figure 23.

As mentioned above, the simulations were performed randomly over the whole range of test speeds and braking manoeuvres. In the following, the errors derived from the braking system parameters are shown in comparison with the empirical data. Table 5 shows the target values for braking type and *q_t_* for the two pressure sensors, the data simulated by ANN for the above parameters and the error of these values compared to the target data.

Braking types 1, 2 and 3 correspond to maintained, progressive and emergency braking, respectively.

Table 6 shows the *vfill_t_* target values for the two pressure sensors, the ANN-simulated data for these parameters and the error of these values against the target data.

Table 7 shows the mean error obtained for the parameters *q_t_*, *vfill_t_* and the type of braking for each type of braking.

Table 8 shows the standard deviation associated with the above mean values.

## 6. Validation of Results against Direct Sensor Readings

As mentioned in Section 3.3, the data collected directly by the sensors were treated to work with them; however, in order to incorporate the proposed estimation system in a real vehicle, it is necessary to interpret the results under the same conditions in which they were taken during the experimental tests.

This section will show the comparison between the values of the simulations carried out by ANN and the empirical values obtained by the sensors during the experimental tests.

The values estimated by ANN are represented in the time in which the system estimates that braking will occur. The braking time is not an output layer value but can be calculated using the simulated values for *q_t_* and *vfill_t_*.

**Figure 24:**
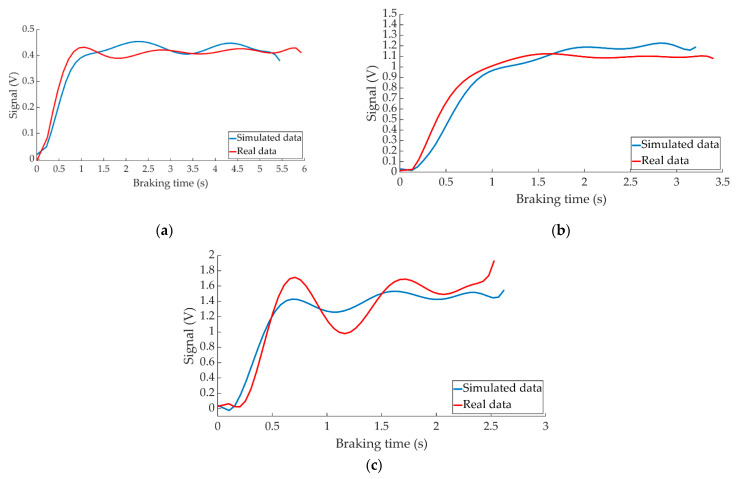
Comparison between the ANN-simulated data and actual data collected by the right pressure sensor for braking at a speed of 70 km/h for the type: (**a**) maintained, (**b**) progressive and (**c**) emergency.

Figure 24 shows the comparison between the data provided by ANN when simulating braking at a test speed of 70 km/h for the three types of braking and the data collected by the right pressure sensor during these tests.

**Figure 25:**
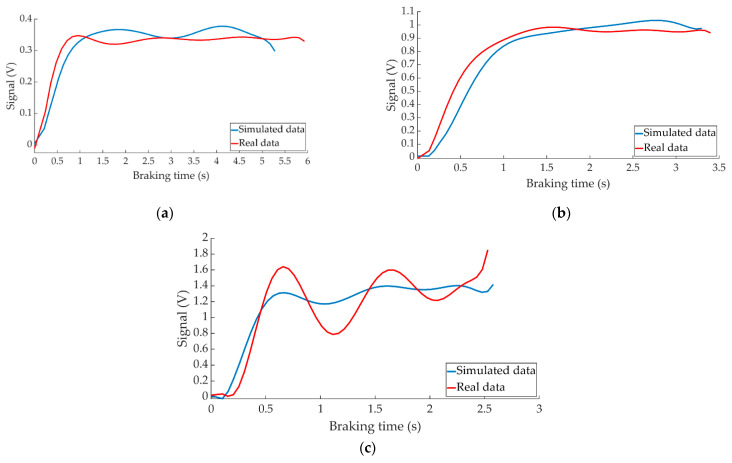
Comparison between the ANN-simulated data and actual data collected by the left pressure sensor for braking at a speed of 70 km/h for the type: (**a**) maintained, (**b**) progressive and (**c**) emergency.

Figure 25 shows the behaviour of the left pressure sensor during braking at 70 km/h for the three types of braking together with the system’s estimation for these manoeuvres.

## 7. Discussion

In the work presented here, an automatic braking system has been proposed. Knowing how the braking system behaves depending on the braking pressure the system will decide, based on the braking distance and the vehicle speed, how to execute the braking manoeuvre.

As for the readings that have been obtained from the pressure sensors during the experiments, Table 2, Table 3 and Table 4 show the increasing average of the values recorded by each sensor due to the increase in speed for the three types of braking. For the same test speed, the minimum values collected correspond to maintained type braking, followed by progressive type braking, and finally, the maximum values are those recorded for emergency braking.

Of the possible network structures that exist, an MLP has been chosen for the design of the estimator, which is a type of feed-forward network. This choice is due to the fact that the data used for the training does not have long dependencies. Different network models have been proposed depending on the number of neurons in the output layer (37, 57, 97, 137, 177 and 217); the number of neurons in the hidden layer (10, 20, 30, 40 and 50) and the type of training (LM, BR and SCG). The best result was obtained by the network with 20 neurons in the hidden layer, 217 neurons in the output layer and BR training. This network presented the highest R^2^ value and the lowest MSE value.

Simulations have been carried out to evaluate the performance of the system designed based on ANN. These simulations show the ability of the proposal to obtain accurate values of the parameters that have been defined to characterise the braking of a vehicle and the type of braking. As can be seen in Table 7, the mean error of the parameter estimating the braking capacity (*q_t_*) of all simulations was 1.345 and 1.946% for the right pressure sensor and left pressure sensor, respectively. The mean error of the parameter estimating the braking intensity (*vfill_t_*) of all simulations was 3.959 and 3.775% for the right pressure sensor and left pressure sensor, respectively. It is worth mentioning that the braking type estimations contain the highest error, mainly for maintained braking (23.024%). It has been found that for the emergency braking type the system does not exceed the braking type set in the training, which offers a higher degree of safety and comfort because the braking system will be controlled from the safety side and less aggressively.

In all cases, the comparative curves between the data obtained from the simulations and the data collected by the pressure sensors during the experiments show that both follow the same behaviour patterns (see Figure 24 and Figure 25).

Based on the results obtained, it can be confirmed that the proposed method can accurately estimate the braking parameters. However, there is still a lot of research to be done. The experiments are limited by the fact that female drivers could not be involved. For future work, female drivers will be recruited to participate in the tests in order to improve this research. Future work needs to be carried out with different sensors, different manoeuvres, different road surfaces, determining weather conditions, multiple trajectories, different vehicles, etc. Other estimation methods such as Fuzzy Logic, Kalman filter or H-Infinity filter can also be used. It would be also interesting to design ANN models with two or more hidden layers.

## 8. Conclusions

A vehicle has been instrumented with pressure sensors incorporated in the independent hydraulic braking circuits of the front wheels, thermocouple in the brake disc and load cell installed on the brake pedal. The effectiveness of these sensors has been demonstrated to take measurements with high precision during dynamic tests. A GPS receiver has also been installed in the vehicle to obtain additional information during tests.

Experimental tests have been carried out in which the driver performs, following a straight path, a series of braking manoeuvres with the vehicle until it stops. Three types of braking are proposed: maintained, progressive and emergency braking, which cover all possible driving circumstances in which the braking system may be involved. These manoeuvres are repeated for a range of test speeds from 20 to 80 km/h in 10-km/h increments. Each test is characterised by the combination of test speed and type of braking.

To analyse the data collected by the pressure sensors during the experimental tests and in order to operate with them homogeneously in the design of the estimator, new parameters were defined: *q_t_*, *vfill_t_*, *q_v_* and *vfill_v_*. Each of these indicators provided relevant information to characterise vehicle braking.

An ANN estimator has been designed to simulate the real values collected by the sensors in order to characterise the braking of a vehicle and to be used in real driving situations. The estimator uses the vehicle speed and the available braking distance as input data and returns, as output data, the pressure to be applied to the braking system over time, as well as the type of braking to be performed, thus achieving the objective set for this research.

## Figures and Tables

**Table 1:** Tests carried out by different drivers.

Test Speed (km/h)	Type of Braking
20	Maintained braking Progressive braking Emergency braking
30	Maintained braking Progressive braking Emergency braking
40	Maintained braking Progressive braking Emergency braking
50	Maintained braking Progressive braking Emergency braking
60	Maintained braking Progressive braking Emergency braking
70	Maintained braking Progressive braking Emergency braking
80	Maintained braking Progressive braking Emergency braking

**Table 2:** Data from variables analysed for each test speed for maintained braking.

Speed (km/h)	Sensor	Maximum	Minimum	Average	Standard Deviation
20	Right pressure (V)	0.618	0.206	0.410	0.153
	Left pressure (V)	0.573	0.184	0.377	0.145
	Braking time (s)	3.53	1.25	2.118	0.697
	Braking distance (m)	10.949	4.031	6.629	2.105
30	Right pressure (V)	0.792	0.264	0.458	0.173
	Left pressure (V)	0.734	0.220	0.419	0.163
	Braking time (s)	3.73	1.7	2.901	0.612
	Braking distance (m)	19.215	8.581	14.292	3.092
40	Right pressure (V)	0.799	0.328	0.549	0.164
	Left pressure (V)	0.740	0.293	0.507	0.156
	Braking time (s)	4.71	1.98	3.3	0.699
	Braking distance (m)	31.757	12.136	21.376	4.967
50	Right pressure (V)	1.165	0.411	0.643	0.225
	Left pressure (V)	1.082	0.375	0.594	0.211
	Braking time (s)	5.44	2.45	3.847	0.884
	Braking distance (m)	40.604	17.194	29.967	7.311
60	Right pressure (V)	1.530	0.519	0.774	0.351
	Left pressure (V)	1.388	0.478	0.709	0.317
	Braking time (s)	5.5	2.75	3.917	0.751
	Braking distance (m)	49.273	24.040	37.255	6.631
70	Right pressure (V)	1.586	0.584	0.803	0.279
	Left pressure (V)	1.481	0.536	0.739	0.262
	Braking time (s)	5.93	2.34	4.259	0.885
	Braking distance (m)	61.163	26.138	45.701	9.021
80	Right pressure (V)	1.679	0.652	0.942	0.374
	Left pressure (V)	1.596	0.595	0.868	0.353
	Braking time (s)	6.23	3	4.426	0.869
	Braking distance (m)	76.692	38.014	54.596	10.266

**Table 3:** Data from variables analysed for each test speed for progressive braking.

Speed (km/h)	Sensor	Maximum	Minimum	Average	Standard Deviation
20	Right pressure (V)	0.720	0.334	0.502	0.118
	Left pressure (V)	0.670	0.312	0.466	0.110
	Braking time (s)	2.6	1.58	2.029	0.357
	Braking distance (m)	8.075	5.505	6.423	0.841
30	Right pressure (V)	0.929	0.418	0.639	0.150
	Left pressure (V)	0.864	0.391	0.595	0.141
	Braking time (s)	3.09	1.88	2.323	0.348
	Braking distance (m)	15.135	8.688	11.378	1.915
40	Right pressure (V)	1.177	0.540	0.817	0.222
	Left pressure (V)	1.066	0.502	0.755	0.200
	Braking time (s)	3.3	2.07	2.568	0.398
	Braking distance (m)	24.314	12.955	17.186	3.197
50	Right pressure (V)	1.349	0.571	0.959	0.255
	Left pressure (V)	1.259	0.526	0.888	0.233
	Braking time (s)	4.12	2.27	2.845	0.522
	Braking distance (m)	33.805	18.447	23.480	4.075
60	Right pressure (V)	1.597	0.641	1.044	0.342
	Left pressure (V)	1.488	0.599	0.966	0.309
	Braking time (s)	3.87	2.4	3.196	0.545
	Braking distance (m)	41.490	22.292	31.409	5.698
70	Right pressure (V)	1.983	0.731	1.265	0.450
	Left pressure (V)	1.701	0.702	1.161	0.391
	Braking time (s)	4.5	2.18	3.377	0.714
	Braking distance (m)	49.581	23.706	37.905	8.934
80	Right pressure (V)	1.992	0.807	1.379	0.404
	Left pressure (V)	1.814	0.748	1.243	0.329
	Braking time (s)	4.58	2.52	3.415	0.596
	Braking distance (m)	58.596	26.537	43.271	9.542

**Table 4:** Data from variables analysed for each test speed for emergency braking.

Speed (km/h)	Sensor	Maximum	Minimum	Average	Standard Deviation
20	Right pressure (V)	1.871	0.759	1.286	0.479
	Left pressure (V)	1.749	0.701	1.197	0.446
	Braking time (s)	1.15	0.64	0.952	0.153
	Braking distance (m)	4.148	2.321	3.202	0.613
30	Right pressure (V)	1.879	1.01	1.471	0.374
	Left pressure (V)	1.786	0.937	1.374	0.321
	Braking time (s)	1.5	1	1.266	0.132
	Braking distance (m)	8.337	5.004	6.176	0.947
40	Right pressure (V)	1.958	0.997	1.687	0.305
	Left pressure (V)	1.894	0.904	1.527	0.278
	Braking time (s)	2.01	1.28	1.607	0.218
	Braking distance (m)	15.594	8.649	10.494	2.198
50	Right pressure (V)	1.986	1.235	1.737	0.235
	Left pressure (V)	1.942	1.122	1.635	0.244
	Braking time (s)	2.04	1.71	1.887	0.092
	Braking distance (m)	17.149	13.480	14.848	1.159
60	Right pressure (V)	2.014	1.476	1.875	0.149
	Left pressure (V)	1.946	1.360	1.709	0.156
	Braking time (s)	3.01	1.98	2.4	0.266
	Braking distance (m)	28.524	17.495	21.891	3.187
70	Right pressure (V)	2.245	1.768	1.999	0.148
	Left pressure (V)	2.055	1.637	1.798	0.122
	Braking time (s)	2.67	2.36	2.523	0.098
	Braking distance (m)	27.838	24.990	26.264	1.103
80	Right pressure (V)	2.351	1.312	2.001	0.244
	Left pressure (V)	2.164	1.155	1.785	0.251
	Braking time (s)	2.93	2.51	2.677	0.121
	Braking distance (m)	39.404	28.348	32.543	3.523

**Table 5:** Target and simulated values of braking type (Tb), right pressure sensor *q_t_* (pr), left pressure sensor *q_t_* (pl) and error.

Test Speed (km/h)	Tb Target	Tb ANN	Error Tb (%)	*q_t_* pr Target (−)	*q_t_* pl Target (−)	*q_t_* pr ANN (−)	*q_t_* pl ANN (−)	Error *q_t_* pr (%)	Error *q_t_* pl (%)
20	1	1.198	19.790	61.666	55.066	62.325	51.528	1.068	6.426
30	1	1.267	26.690	83.841	76.920	83.896	74.716	0.066	2.866
40	1	1.339	33.870	144.547	132.640	143.452	132.713	0.758	0.055
50	1	1.268	26.780	176.539	161.390	175.466	163.674	0.608	1.415
60	1	1.273	27.340	196.110	176.927	200.004	182.471	1.986	3.134
70	1	0.934	6.640	212.132	189.399	210.982	189.003	0.542	0.209
80	1	0.799	20.060	256.899	224.802	257.644	224.558	0.290	0.109
20	2	1.839	8.075	68.920	64.861	67.529	63.509	2.018	2.084
30	2	2.175	8.755	91.568	85.262	91.926	85.583	0.391	0.376
40	2	2.345	17.255	149.352	139.339	149.797	138.186	0.298	0.827
50	2	1.749	12.560	174.557	162.725	174.978	162.466	0.241	0.160
60	2	1.931	3.475	256.852	237.602	242.019	228.535	5.775	3.816
70	2	1.899	5.040	304.726	284.060	304.717	283.668	0.003	0.138
80	2	1.872	6.425	351.586	315.649	347.118	316.410	1.271	0.241
20	3	2.302	23.283	84.704	80.895	79.247	73.292	6.443	9.399
30	3	2.694	10.213	121.171	108.838	122.686	114.119	1.250	4.852
40	3	2.502	16.610	153.193	141.558	149.638	138.841	2.321	1.920
50	3	2.850	5.010	218.325	193.090	216.281	194.676	0.936	0.821
60	3	2.656	11.470	284.893	256.028	282.748	258.274	0.753	0.877
70	3	2.560	14.673	330.512	289.901	328.298	291.454	0.670	0.536
80	3	2.723	9.220	367.199	333.607	365.160	331.577	0.555	0.609

**Table 6:** Target and simulated values of *vfill_t_* of the right pressure sensor (pr), *vfill_t_* of the left pressure sensor (pl) and error.

Test Speed (km/h)	Tb	*vfill_t_* pr Target (−)	*vfill_t_* pl Target (−)	*vfill_t_* pr ANN (−)	*vfill_t_* pl ANN (−)	Error *vfill_t_* pr (%)	Error *vfill_t_* pl (%)
20	1	0.453	0.420	0.461	0.430	1.674	2.306
30	1	0.448	0.408	0.412	0.384	8.110	5.841
40	1	0.315	0.284	0.307	0.279	2.699	1.759
50	1	0.354	0.324	0.339	0.307	4.191	5.244
60	1	0.484	0.437	0.478	0.437	1.306	0.081
70	1	0.358	0.319	0.387	0.358	8.295	12.057
80	1	0.412	0.361	0.405	0.370	1.736	2.456
20	2	0.428	0.403	0.433	0.404	1.150	0.332
30	2	0.430	0.400	0.453	0.424	5.463	5.962
40	2	0.692	0.645	0.745	0.694	7.579	7.675
50	2	0.619	0.577	0.626	0.580	1.099	0.548
60	2	0.895	0.828	0.847	0.774	5.392	6.545
70	2	0.896	0.835	0.950	0.860	5.952	2.960
80	2	0.623	0.581	0.616	0.568	1.011	2.310
20	3	0.538	0.510	0.530	0.495	1.475	2.893
30	3	0.927	0.817	0.892	0.829	3.795	1.418
40	3	0.876	0.747	0.853	0.793	2.644	6.233
50	3	1.200	1.061	1.110	1.021	7.485	3.792
60	3	1.217	1.094	1.170	1.065	3.942	2.663
70	3	1.306	1.146	1.251	1.129	4.254	1.454
80	3	1.429	1.298	1.373	1.236	3.898	4.752

**Table 7:** Mean error in the results of the braking type, *q_t_* and *vfill_t_* simulations of the pressure sensors.

Tb	Mean Error Tb (%)	Mean Error *q_t_* pr (%)	Mean Error *q_t_* pl (%)	Mean Error *vfill_t_* pr (%)	Mean Error *vfill_t_* pl (%)
1	23.024	0.760	2.030	4.002	4.249
2	8.798	1.428	1.092	3.949	3.762
3	12.926	1.847	2.716	3.927	3.315
Total	14.916	1.345	1.946	3.959	3.775

**Table 8:** Standard deviation in the results of the brake type, *q_t_* and *vfill_t_* simulations of pressure sensors.

Tb	Standard Deviation Tb (%)	Standard Deviation *q_t_* pr (%)	Standard Deviation *q_t_* pl (%)	Standard Deviation *vfill_t_* pr (%)	Standard Deviation *vfill_t_* pl (%)
1	8.675	0.629	2.328	3.024	3.980
2	4.731	2.044	1.384	2.773	2.965
3	5.913	2.113	3.317	1.846	1.755
Total	6.439	1.595	2.343	2.548	2.900
